# Regulatory Role of the Transcription Factor Twist1 in Cancer-Associated Muscle Cachexia

**DOI:** 10.3389/fphys.2020.00662

**Published:** 2020-06-23

**Authors:** Mohammed S. Razzaque, Azeddine Atfi

**Affiliations:** ^1^Department of Pathology, Lake Erie College of Osteopathic Medicine, Erie, PA, United States; ^2^Department of Pathology, Virginia Commonwealth University, Richmond, VA, United States

**Keywords:** activin A, twist1, MuRF1, atrogin1, muscle atrophy

## Abstract

Muscle cachexia is a catabolic response, usually takes place in various fatal diseases, such as sepsis, burn injury, and chronic kidney disease. Muscle cachexia is also a common co-morbidity seen in the vast majority of advanced cancer patients, often associated with low quality of life and death due to general organ dysfunction. The triggering events and underlying molecular mechanisms of muscle wasting are not yet clearly defined. Our recent study has shown that the ectopic expression of Twist1 in muscle progenitor cells is sufficient to drive muscle structural protein breakdown and attendant muscle atrophy, reminiscent of muscle cachexia. Intriguingly, muscle Twist1 expression is highly induced in cachectic muscles from several mouse models of pancreatic ductal adenocarcinoma (PDAC), raising the interesting possibility that Twist1 may mediate PDAC-driven muscle cachexia. Along these lines, both genetic and pharmacological inactivation of Twist1 function was highly significant at protecting against cancer cachexia, which translated into a significant survival benefit in the experimental PDAC animals. From a translational perspective, elevated expression of Twist1 is also detected in cancer patients with severe muscle wasting, implicating a role of Twist1 in cancer cachexia, and further providing a possible target for therapeutic attenuation of cachexia to improve cancer patient survival. In this article, we will briefly summarize how Twist1 acts as a master regulator of tumor-induced cachexia, and discuss the relevance of our findings to muscle wasting diseases in general. The mechanism of decreased muscle mass in various catabolic conditions is thought to rely on similar pathways, and, therefore, Twist1-induced cancer cachexia may benefit diverse groups of patients with clinical complications associated with loss of muscle mass and functions, beyond the expected benefits for cancer patients.

## Introduction

Cachexia, a hypercatabolic state, is a commonly encountered adverse effect of cancer, and markedly impairs the quality of life by harmfully impacting both the physical and psychosocial behaviors. An international consensus reached in 2011 explained cancer cachexia as a multifactorial condition with continuing skeletal muscle loss that is not reversible by standard nutritional support, ultimately leading to functional impairment (Fearon et al., 2011). A skeletal muscle index <7.26 kg/m^2^ in males and <5.45 kg/m^2^ in females is considered as cachexia. Of relevance, the majority of patients with pancreatic tumors display signs of cachexia at the time of diagnosis (Fearon et al., 2006). Even the overweight pancreatic cancer patients develop cachexia (sarcopenic obesity), and cachexia hidden in obesity causes extensive muscle loss, with the pathological accumulation of adipose tissue that influences the overall survival of the patients (Tan et al., 2009). In cancer patients, cachexia is a progressive process that evolves through various stages – from pre-cachexia to cachexia to refractory cachexia (irreversible stage). It is important to find measures to reverse from cachexia into pre-cachectic stages to provide relief to the affected patients. Besides cancer, cachexia can also occur in a wide range of disorders, ranging from infections to chronic kidney diseases to cerebrovascular diseases, including stroke and chronic obstructive pulmonary diseases (Reid et al., 2013; Scherbakov et al., 2013; Morley, 2014). Severe muscle wasting or cachexia is noted in up to 75% of chronic kidney disease patients undergoing hemodialysis treatment (Mak et al., 2011).

The underlying mechanism of cachexia, in tumor and other catabolic disorders, are not yet clearly understood. Various catabolic conditions are associated with altered expression and regulations of transcription factors and nuclear cofactors that induce a specific group of genes, which are believed to execute the final steps of muscle atrophy. Two muscle-specific ubiquitin ligases, MuRF1 and Atrogin1/MAFbx, are essential for the degradation of muscle proteins, including myosin heavy chain (MHC) and eukaryotic initiation factor 3f (Elf-3f) (Clarke et al., 2007; Lagirand-Cantaloube et al., 2008). Transcription factors, FOXO1 is an important regulator of muscle atrophy, and is shown to be affected by sepsis and elevated levels of glucocorticoids (Stitt et al., 2004; Crossland et al., 2008; Waddell et al., 2008; Reed et al., 2012; Xu et al., 2012; Huynh et al., 2013). It is a key regulator of genes involved in muscle wasting, including Atrogin-1 and MuRF1 (Li et al., 2007; Leger et al., 2009; Pomies et al., 2016). FOXO1 also regulates genes involved in the autophagy-lysosomal proteolytic pathway (Sengupta et al., 2009; Milan et al., 2015). MyoD is a muscle-specific transcription factor that regulates muscle cell differentiation (Davis et al., 1987). Recently, MyoD-induced muscle cell differentiation is shown to be mediated by Twist1 through miR-206 (Koutalianos et al., 2015). Our recent studies suggest that the transcription factor Twist1 is also actively involved in the regulation of cancer-induced muscle wasting presumably owing to its ability to induce the expression of MuRF1 and Atrogin1, thereby causing muscle protein degradation and attendant muscle cachexia (Parajuli et al., 2018).

Of particular clinical importance, developing effective treatments to curb cachexia and muscle wasting disorders are essential for improving the quality of health and survival of the cancer patients and beyond. Tumor necrosis factor-alpha (TNF-α), interleukin 1 (IL-1), interleukin 6 (IL-6) and interferon-gamma (IFN-γ) are the main cytokines that are thought to be involved in the evolvement of cachexia, in general (Fong et al., 1989; Strassmann et al., 1993; Kayacan et al., 2006; White, 2017). However, clinically targeting these cytokines showed mixed results. For instance, in a clinical trial, infliximab (anti-TNF-α monoclonal antibody) showed no improvement of cachexia in cancer patients (Jatoi et al., 2010). In contrast, treating tumor patients with a humanized monoclonal anti-IL-6 antibody increased hemoglobin levels and reduced muscle wasting (Rigas et al., 2010).

## Twist1

Twist was initially identified in Drosophila (Thisse et al., 1987). Later, Twist isoforms have been identified in humans and mice (Wolf et al., 1991; Wang et al., 1997). Twist1 is a member of the basic helix-loop-helix (bHLH) transcription factor family that controls the activity of genes essential for embryogenesis and organogenesis (el Ghouzzi et al., 1997; Wang et al., 1997; Pan et al., 2009). Human and mouse Twist1 proteins share a very high amino acid sequence identity (96%). The Twist1 protein is involved in the generation and maturation of cells that eventually form the musculoskeletal system. Notably, during development, Twist proteins transiently inhibit Runx2 function, causing osteoblast-specific gene expression that leads to osteoblast differentiation (Bialek et al., 2004). Of relevance, human Twist1 is highly expressed in fetal myoblasts, and its level diminishes in the later stages of development (Koutsoulidou et al., 2011). Mutations in the *TWIST1* gene in human is associated with craniosynostosis (premature closure of the sutures between the bones of the skull), as noted in the Saethre-Chotzen syndrome-affected individuals (Howard et al., 1997). Heterozygous *Twist1* knockout mice showed craniofacial and limb abnormalities, mimicking clinical features of Saethre-Chotzen syndrome patients. Of note, homozygous Twist1 knockout mice were embryonically lethal, suggesting a crucial role of this gene in embryonic survival and development (Chen and Behringer, 1995). In adult mice, Twist1 is expressed in a limited number of tissues, including fibroblasts of the mammary glands and dermal papilla cells of the hair follicles (Xu et al., 2013). Consequently, inducible knockout of *Twist1* in adult mice did not affect their overall health and viability, implicating a more important role of Twist1 during early development than in adult life (Xu et al., 2013).

Studies using breast cancer cell lines have shown an important role of Twist1 in epithelial-to-mesenchymal transformation, intravasation and metastasis (Xu et al., 2017); more importantly, genetically ablating the Twist1 function effectively inhibited breast tumor cell intravasation and lung metastasis (Xu et al., 2017). In a similar line of study, Twist1 overexpression has shown to be associated with the progression of several human malignant tumors, including pancreatic ductal adenocarcinoma (PDAC) (Lee and Bar-Sagi, 2010; Qin et al., 2012).

The role of Twist1 in myogenesis is not clear. In Drosophila, Twist has been shown to enhance myogenesis, while in mouse myoblasts (C2C12) and human embryonic stem cells (embryoid bodies), Twist1 has shown to inhibit muscle cell differentiation (Hebrok et al., 1994; Rohwedel et al., 1995; Cao et al., 2008; Koutsoulidou et al., 2011). Moreover, overexpression of Twist1 reverses the process of muscle cell differentiation (Hjiantoniou et al., 2008; Mastroyiannopoulos et al., 2013). Recently, we have shown that induction of Twist1 is also related to muscle cachexia during the progression of cancer (Parajuli et al., 2018).

## Twist1 Activation and Cancer-Induced Cachexia

Studies have shown that numerous hormones, cytokines, and tumor-derived factors play key roles in the initiation and propagation of cancer cachexia by involving several major intracellular signaling systems (Benny Klimek et al., 2010). ActRIIB is a high-affinity activin type two receptor that facilitates the signaling of various factors, including myostatin, and activin (Lee and McPherron, 2001; Souza et al., 2008). Induced expression of activin could cause cachexia in tumor-free mice (Chen et al., 2014). Myostatin is a secreted protein of the TGF-β family, which is mostly expressed in skeletal muscle, including muscle progenitor satellite cells. In a mouse model of pancreatic cancer-induced cachexia, therapeutic reduction of TGF-β resulted in reduced cachexia and increased survival (Greco et al., 2015). Furthermore, increased signaling activity through ActRIIB pathway has shown to be involved in both tumorigenesis and cancer-induced cachexia (Wildi et al., 2001; Costelli et al., 2008; Zhou et al., 2010). Intriguingly, blocking the bioactivities of ActRIIB has been shown to reverse cancer-induced cachexia and cardiac atrophy, and this response resulted in the extended lifespan of the experimental animals even without reducing the tumor growth (Zhou et al., 2010).

As mentioned, two muscle-specific ubiquitin ligases, MuRF1, and Atrogin1/MAFbx are essential to muscle protein degradation, including MHC and Elf-3f (Clarke et al., 2007; Lagirand-Cantaloube et al., 2008). Myostatin can induce the expression of MuRF1 and Atrogin1/MAFbx as well as Twist1 (Parajuli et al., 2018), and genetic inactivation of myostatin has shown to protect against cancer-induced cachexia (Gallot et al., 2014). Selectively inducing Twis1 in mesenchymal stem cells using mouse genetics tools caused severe hypotrophy of the skeletal muscle (Parajuli et al., 2018). Interestingly, when similar *in vivo* studies were conducted on muscle progenitor cells (satellite cells), overexpression of Twist1 resulted in the loss of muscle mass in adult mice. Morphological analysis of the Twist1 overexpressing atrophic muscle showed markedly reduced myofiber diameters, as compared to the control animals, clearly demonstrating the *in vivo* role of Twist1 hyperactivity in muscle atrophy (Parajuli et al., 2018).

## Therapeutic Potential of Twist1 in Cancer

In a healthy-weight individual, skeletal muscle comprises almost 40% of total human body mass (Rolfe and Brown, 1997). Studies have shown that patients with pancreatic cancer have often developed severe cachexia, which is associated with substantial weight loss and skeletal muscle atrophy. Noteworthy, conventional nutritional support cannot fully reverse the loss of muscle function in these patients. Almost one-third of cachectic patients develop severe respiratory muscle dysfunction, causing death due to cardiopulmonary failure in pancreatic cancer patients (Bachmann et al., 2008). In experimental models of pancreatic cancer, reducing cachexia can improve overall survival, despite persistent tumor growth, suggesting that cachexia is an important determinant of survival in tumor patients (Tisdale, 2010).

**FIGURE 1:**
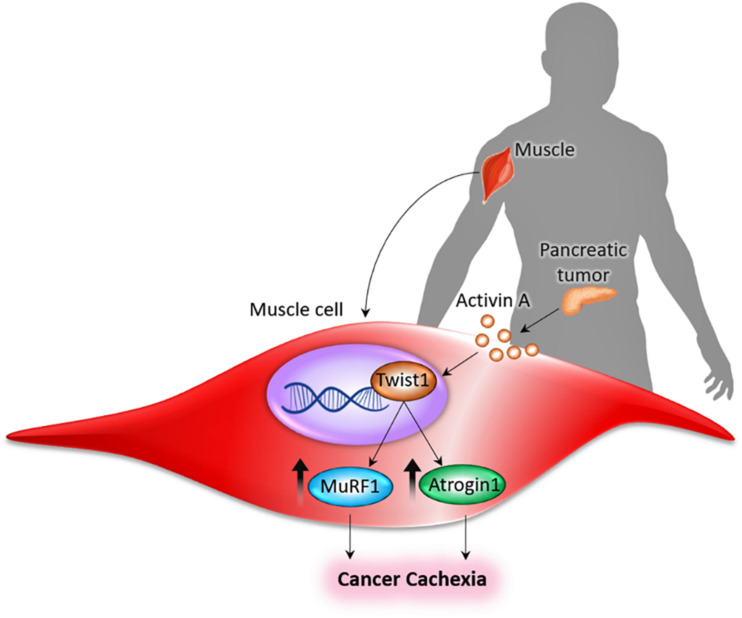
Pancreas-muscle axis. Tumor-derived Activin A acts on the muscle to upregulate the expression of Twist1, which in turn induces the synthesis of the muscle-specific ubiquitin ligases (MuRF1 and Atrogin1), thereby causing cancer cachexia by facilitating muscle protein degradation (Parajuli et al., 2018).

Our studies have shown that tumor-derived Activin A acts on the muscle to upregulate the expression of Twist1, which in turn induces the synthesis of the muscle-specific ubiquitin ligases, MuRF1 and Atrogin1, thereby causing muscle cachexia by facilitating muscle protein degradation (Figure 1; Parajuli et al., 2018). In experimental studies, serum activin levels correlated with PDAC-induced cachexia and eventual mortality (Zhong et al., 2019). In the murine model of PDAC-induced cachexia, activins (activin-βA, or Inhba) are expressed, both in tumor cells and tumor stromal cells. Treatment with an activin inhibitor in a murine model of PDAC-induced cachexia reduced weight loss and cachexia with the resultant effect being prolonged survival (Zhong et al., 2019). Moreover, using the pharmacological drug JQ1, a small molecule that suppresses Twist1 activity by blunting its binding to MuRF1 and Atrogin1 promoters, muscle cachexia could be reversed in PDAC mice deleted of Twist1, indicating that inhibition of Twist1 activity in muscle in indispensable for preventing muscle cachexia. Treatment with JQ1 prevented weight loss, which was associated with increased muscle mass and myofiber size, in turn resulting in improved muscle function and better survival of the PDAC-induced cachectic mice (Parajuli et al., 2018). It is important to emphasize that the apparent survival benefit of these experimentally induced tumor models (due to suppression of Twist1 activity) was mostly related to the reversal of muscle cachexia, and not due to shrinkage of tumor (Parajuli et al., 2018). These *in vivo* observations suggest that Twist1 could be a therapeutic target to reduce muscle mass loss in tumor and other chronic debilitating diseases, not only to improve quality of life, but also to increase disease-free survival.

In mice with chronic kidney disease, a two- to three-fold increase in myostatin expression was detected in muscle (Zhang et al., 2011); after 7 days of treatment with the anti-myostatin peptibody, muscle weights in mice with chronic kidney disease was significantly greater than those in vehicle-treated chronic kidney disease mice. Such gain of muscle mass was also reflected in the body weight gain of mice with chronic kidney disease that were treated with the anti-myostatin peptibody (Zhang et al., 2011). Furthermore, the elevated level of activin A was detected in various tissues in mice with chronic kidney disease (Williams et al., 2018). Interestingly, experimentally induced chronic kidney disease animals also showed higher expression of Atrogin-1 and MuRF-1 (Avin et al., 2016). Whether such an increase in the expression of activin A in chronic kidney disease leads to the activation of Twist1 to induce the expression of Atrogin-1 and MuRF-1 needs further studies.

Given the similarities in the general mechanisms governing muscle cachexia, one would surmise that the development of a therapeutic strategy to reduce the disease burden associated with reduced muscle function and cachexia would also benefit patients beyond tumor (Table 1). For instance, in chronic kidney disease patients undergoing hemodialysis treatment, the stable weight patients have better survival than those with weight loss (Villain et al., 2015). Of clinical importance, muscle wasting or cachexia occurs in up to 75% of chronic kidney disease patients on hemodialysis (Mak et al., 2011). Despite such widespread occurring of muscle wasting and its adverse impact on the survival of chronic kidney disease patients, there is no selective and effective clinical treatment of cachexia in patients with chronic kidney disease. Reducing the abundance of MuRF1 and atrogin-1 in skeletal muscles of the tumor and chronic diseases through targeting upstream regulators would likely to attenuate muscle cachexia (Zhang et al., 2018).

**TABLE 1:** A partial list of the disorders associated with muscle wasting.

• Aging
• Anorexia nervosa
• Burns
• Cancer
• Chronic kidney disease
• Chronic obstructive pulmonary disease
• Congestive heart failure
• Cystic fibrosis
• Dermatomyositis
• Guillain-Barre Syndrome
• Lack of physical activity
• Long-term corticosteroid therapy
• Malnutrition (Kwashiorkor)
• Multiple sclerosis
• Osteoarthritis
• Peripheral neuropathy
• Polio (viral disease)
• Poliomyelitis
• Rheumatoid arthritis
• Sepsis
• Spinal cord injury

## Conclusion

Cachexia occurs in many end-stage illnesses, including cancers, chronic kidney diseases, chronic cardiac diseases, chronic obstructive pulmonary diseases, chronic liver diseases, severe burns, HIV infections, rheumatoid arthritis, and aging (Mattox, 2017; von Haehling et al., 2017; Baracos et al., 2018; Scicchitano et al., 2018; Thakur et al., 2018). Roughly, 30% of patients with chronic lung, liver, heart or kidney diseases develop cachexia, while around 50% of cancer patients develop that syndrome, either as a direct consequence of the disease itself or as a consequence of treatment. Since cachexia cannot always be reversed by nutritional supplements, its underlying mechanism is different than that of an eating disorder, such as anorexia. Moreover, cachexia usually affects the loss of the muscular component of the body, while starvation initially initiates the loss of fat mass (Morley et al., 2006). The overall devastating impact of cachexia on patients with chronic diseases can not only reduce physical activities and quality of life but more importantly, can shorten survival (Farkas et al., 2013). Hence, developing effective treatments to reduce the progression of cachexia and muscle wasting disorders are essential clinical need to reduce disease burden and improve the quality of life and survival of the cancer patients and beyond. Twist1 promotes epithelial-mesenchymal transition, invasion, metastasis, and chemotherapy resistance in cancer cells and thus is a potential target for cancer therapy (Kang and Massague, 2004; Vernon and LaBonne, 2004; Yang et al., 2004; Lee et al., 2006; Yuen et al., 2007). Our recent identification of Twist1 as a master regulator of tumor-induced cachexia provides a promising therapeutic target to attenuate cachexia to improve cancer patient survival. In fact, pharmacological inactivation of the Twist1 function showed promising effects of protecting cancer-induced cachexia, with a significant survival benefit in the experimental model of pancreatic carcinoma (Parajuli et al., 2018). Based on the inducible Twist1 knockout mice studies, it appears that Twist1 has rather a non-essential role in adult animals (Xu et al., 2013), and therefore, targeting Twist1 to manipulate tumor-induced cachexia would be a suitable drug target that is likely to exert minimal advert effects in adult patients. Further studies are needed to determine the effects of suppressing Twist1 function in muscle wasting diseases, in general.

## Author Contributions

MR and AA outlined and drafted the manuscript. Both authors contributed to the article and approved the submitted version.

## Conflict of Interest

The authors declare that the research was conducted in the absence of any commercial or financial relationships that could be construed as a potential conflict of interest.

## Abbreviations

Atrogin1/MAFbx: atrogin1/muscle atrophy F-box
bHLH: basic helix-loop-helix Runx2
Elf-3f: eukaryotic initiation factor 3f FOXO1
HIV: human immunodeficiency viruses
IL-1: interleukin 1
IL-6: interleukin 6
INF- γ: interferon-gamma
MHC: myosin heavy chain
miR-206: microRNA-206
MuRF1: muscle RING finger 1
PDAC: pancreatic ductal adenocarcinoma
TGF- β: transforming growth factor- β
TNF- α: tumor necrosis factor-alpha
Twist1: twist family bHLH transcription factor 1.

## References

[B1] Avin K. G. Chen N. X. Organ J. M. Zarse C. O’neill K. Conway R. G. (2016). Skeletal muscle regeneration and oxidative stress are altered in chronic kidney disease. *PLoS One* 11:e0159411. 10.1371/journal.pone.0159411 27486747 PMC4972446

[B2] Bachmann J. Heiligensetzer M. Krakowski-Roosen H. Buchler M. W. Friess H. Martignoni M. E. (2008). Cachexia worsens prognosis in patients with resectable pancreatic cancer. *J. Gastrointest. Surg.* 12 1193–1201. 10.1007/s11605-008-0505-z 18347879

[B3] Baracos V. E. Martin L. Korc M. Guttridge D. C. Fearon K. C. H. (2018). Cancer-associated cachexia. *Nat. Rev. Dis. Primers* 4:17105. 10.1038/nrdp.2017.105 29345251

[B4] Benny Klimek M. E. Aydogdu T. Link M. J. Pons M. Koniaris L. G. Zimmers T. A. (2010). Acute inhibition of myostatin-family proteins preserves skeletal muscle in mouse models of cancer cachexia. *Biochem. Biophys. Res. Commun.* 391 1548–1554. 10.1016/j.bbrc.2009.12.123 20036643

[B5] Bialek P. Kern B. Yang X. Schrock M. Sosic D. Hong N. (2004). A twist code determines the onset of osteoblast differentiation. *Dev. Cell* 6 423–435. 10.1016/s1534-5807(04)00058-9 15030764

[B6] Cao F. Wagner R. A. Wilson K. D. Xie X. Fu J. D. Drukker M. (2008). Transcriptional and functional profiling of human embryonic stem cell-derived cardiomyocytes. *PLoS One* 3:e3474. 10.1371/journal.pone.0003474 18941512 PMC2565131

[B7] Chen J. L. Walton K. L. Winbanks C. E. Murphy K. T. Thomson R. E. Makanji Y. (2014). Elevated expression of activins promotes muscle wasting and cachexia. *FASEB J.* 28 1711–1723. 10.1096/fj.13-245894 24378873

[B8] Chen Z. F. Behringer R. R. (1995). twist is required in head mesenchyme for cranial neural tube morphogenesis. *Genes Dev.* 9 686–699. 10.1101/gad.9.6.686 7729687

[B9] Clarke B. A. Drujan D. Willis M. S. Murphy L. O. Corpina R. A. Burova E. (2007). The E3 Ligase MuRF1 degrades myosin heavy chain protein in dexamethasone-treated skeletal muscle. *Cell Metab.* 6 376–385. 10.1016/j.cmet.2007.09.009 17983583

[B10] Costelli P. Muscaritoli M. Bonetto A. Penna F. Reffo P. Bossola M. (2008). Muscle myostatin signalling is enhanced in experimental cancer cachexia. *Eur. J. Clin. Invest.* 38 531–538. 10.1111/j.1365-2362.2008.01970.x 18578694

[B11] Crossland H. Constantin-Teodosiu D. Gardiner S. M. Constantin D. Greenhaff P. L. (2008). A potential role for Akt/FOXO signalling in both protein loss and the impairment of muscle carbohydrate oxidation during sepsis in rodent skeletal muscle. *J. Physiol.* 586 5589–5600. 10.1113/jphysiol.2008.160150 18818241 PMC2655379

[B12] Davis R. L. Weintraub H. Lassar A. B. (1987). Expression of a single transfected cDNA converts fibroblasts to myoblasts. *Cell* 51 987–1000. 10.1016/0092-8674(87)90585-x 3690668

[B13] el Ghouzzi V. Le Merrer M. Perrin-Schmitt F. Lajeunie E. Benit P. Renier D. (1997). Mutations of the TWIST gene in the Saethre-Chotzen syndrome. *Nat. Genet.* 15 42–46. 8988167 10.1038/ng0197-42

[B14] Farkas J. Von Haehling S. Kalantar-Zadeh K. Morley J. E. Anker S. D. Lainscak M. (2013). Cachexia as a major public health problem: frequent, costly, and deadly. *J. Cachexia Sarcopenia Muscle* 4 173–178. 10.1007/s13539-013-0105-y 23539127 PMC3774921

[B15] Fearon K. Strasser F. Anker S. D. Bosaeus I. Bruera E. Fainsinger R. L. (2011). Definition and classification of cancer cachexia: an international consensus. *Lancet Oncol.* 12 489–495. 10.1016/s1470-2045(10)70218-7 21296615

[B16] Fearon K. C. Voss A. C. Hustead D. S. (2006). Definition of cancer cachexia: effect of weight loss, reduced food intake, and systemic inflammation on functional status and prognosis. *Am. J. Clin. Nutr.* 83 1345–1350. 10.1093/ajcn/83.6.1345 16762946

[B17] Fong Y. Moldawer L. L. Marano M. Wei H. Barber A. Manogue K. (1989). Cachectin/TNF or IL-1 alpha induces cachexia with redistribution of body proteins. *Am. J. Physiol.* 256 R659–R665. 2784290 10.1152/ajpregu.1989.256.3.R659

[B18] Gallot Y. S. Durieux A. C. Castells J. Desgeorges M. M. Vernus B. Plantureux L. (2014). Myostatin gene inactivation prevents skeletal muscle wasting in cancer. *Cancer Res.* 74 7344–7356. 10.1158/0008-5472.can-14-0057 25336187

[B19] Greco S. H. Tomkotter L. Vahle A. K. Rokosh R. Avanzi A. Mahmood S. K. (2015). TGF-beta blockade reduces mortality and metabolic changes in a validated murine model of pancreatic cancer cachexia. *PLoS One* 10:e0132786. 10.1371/journal.pone.0132786 26172047 PMC4501823

[B20] Hebrok M. Wertz K. Fuchtbauer E. M. (1994). M-twist is an inhibitor of muscle differentiation. *Dev. Biol.* 165 537–544. 10.1006/dbio.1994.1273 7958419

[B21] Hjiantoniou E. Anayasa M. Nicolaou P. Bantounas I. Saito M. Iseki S. (2008). Twist induces reversal of myotube formation. *Differentiation* 76 182–192. 10.1111/j.1432-0436.2007.00195.x 17662069

[B22] Howard T. D. Paznekas W. A. Green E. D. Chiang L. C. Ma N. Ortiz De Luna R. I. (1997). Mutations in TWIST, a basic helix-loop-helix transcription factor, in Saethre-Chotzen syndrome. *Nat. Genet.* 15 36–41. 10.1038/ng0197-36 8988166

[B23] Huynh T. Uaesoontrachoon K. Quinn J. L. Tatem K. S. Heier C. R. Van Der Meulen J. H. (2013). Selective modulation through the glucocorticoid receptor ameliorates muscle pathology in mdx mice. *J. Pathol.* 231 223–235. 10.1002/path.4231 23794417 PMC4104819

[B24] Jatoi A. Ritter H. L. Dueck A. Nguyen P. L. Nikcevich D. A. Luyun R. F. (2010). A placebo-controlled, double-blind trial of infliximab for cancer-associated weight loss in elderly and/or poor performance non-small cell lung cancer patients (N01C9). *Lung Cancer* 68 234–239. 10.1016/j.lungcan.2009.06.020 19665818 PMC5951722

[B25] Kang Y. Massague J. (2004). Epithelial-mesenchymal transitions: twist in development and metastasis. *Cell* 118 277–279. 15294153 10.1016/j.cell.2004.07.011

[B26] Kayacan O. Karnak D. Beder S. Gullu E. Tutkak H. Senler F. C. (2006). Impact of TNF-alpha and IL-6 levels on development of cachexia in newly diagnosed NSCLC patients. *Am. J. Clin. Oncol.* 29 328–335. 10.1097/01.coc.0000221300.72657.e0 16891858

[B27] Koutalianos D. Koutsoulidou A. Mastroyiannopoulos N. P. Furling D. Phylactou L. A. (2015). MyoD transcription factor induces myogenesis by inhibiting Twist-1 through miR-206. *J. Cell Sci.* 128 3631–3645. 10.1242/jcs.172288 26272918

[B28] Koutsoulidou A. Mastroyiannopoulos N. P. Furling D. Uney J. B. Phylactou L. A. (2011). Endogenous TWIST expression and differentiation are opposite during human muscle development. *Muscle Nerve* 44 984–986. 10.1002/mus.22241 22102471

[B29] Lagirand-Cantaloube J. Offner N. Csibi A. Leibovitch M. P. Batonnet-Pichon S. Tintignac L. A. (2008). The initiation factor eIF3-f is a major target for atrogin1/MAFbx function in skeletal muscle atrophy. *EMBO J.* 27 1266–1276. 10.1038/emboj.2008.52 18354498 PMC2367397

[B30] Lee K. E. Bar-Sagi D. (2010). Oncogenic KRas suppresses inflammation-associated senescence of pancreatic ductal cells. *Cancer Cell* 18 448–458. 10.1016/j.ccr.2010.10.020 21075310 PMC3397918

[B31] Lee S. J. McPherron A. C. (2001). Regulation of myostatin activity and muscle growth. *Proc. Natl. Acad. Sci. U.S.A.* 98 9306–9311. 10.1073/pnas.151270098 11459935 PMC55416

[B32] Lee T. K. Poon R. T. Yuen A. P. Ling M. T. Kwok W. K. Wang X. H. (2006). Twist overexpression correlates with hepatocellular carcinoma metastasis through induction of epithelial-mesenchymal transition. *Clin. Cancer Res.* 12 5369–5376. 10.1158/1078-0432.ccr-05-2722 17000670

[B33] Leger B. Senese R. Al-Khodairy A. W. Deriaz O. Gobelet C. Giacobino J. P. (2009). Atrogin-1, MuRF1, and FoXO, as well as phosphorylated GSK-3beta and 4E-BP1 are reduced in skeletal muscle of chronic spinal cord-injured patients. *Muscle Nerve* 40 69–78. 10.1002/mus.21293 19533653

[B34] Li H. H. Willis M. S. Lockyer P. Miller N. Mcdonough H. Glass D. J. (2007). Atrogin-1 inhibits Akt-dependent cardiac hypertrophy in mice via ubiquitin-dependent coactivation of Forkhead proteins. *J. Clin. Invest.* 117 3211–3223. 10.1172/jci31757 17965779 PMC2040316

[B35] Mak R. H. Ikizler A. T. Kovesdy C. P. Raj D. S. Stenvinkel P. Kalantar-Zadeh K. (2011). Wasting in chronic kidney disease. *J. Cachexia Sarcopenia Muscle* 2 9–25. 21475675 10.1007/s13539-011-0019-5 PMC3063874

[B36] Mastroyiannopoulos N. P. Antoniou A. A. Koutsoulidou A. Uney J. B. Phylactou L. A. (2013). Twist reverses muscle cell differentiation through transcriptional down-regulation of myogenin. *Biosci. Rep.* 33:e00083. 10.1042/BSR20130068 PMC3848576 24188104

[B37] Mattox T. W. (2017). Cancer cachexia: cause, diagnosis, and treatment. *Nutr. Clin. Pract.* 32 599–606. 10.1177/0884533617722986 28825869

[B38] Milan G. Romanello V. Pescatore F. Armani A. Paik J. H. Frasson L. (2015). Regulation of autophagy and the ubiquitin-proteasome system by the FoxO transcriptional network during muscle atrophy. *Nat. Commun.* 6:6670. 10.1038/ncomms7670 PMC4403316 25858807

[B39] Morley J. E. (2014). Chronic obstructive pulmonary disease: a disease of older persons. *J. Am. Med. Dir. Assoc.* 15 151–153. 24513223 10.1016/j.jamda.2013.12.078

[B40] Morley J. E. Thomas D. R. Wilson M. M. (2006). Cachexia: pathophysiology and clinical relevance. *Am. J. Clin. Nutr.* 83 735–743. 10.1093/ajcn/83.4.735 16600922

[B41] Pan D. Fujimoto M. Lopes A. Wang Y. X. (2009). Twist-1 is a PPARdelta-inducible, negative-feedback regulator of PGC-1alpha in brown fat metabolism. *Cell* 137 73–86. 10.1016/j.cell.2009.01.051 19345188 PMC2688451

[B42] Parajuli P. Kumar S. Loumaye A. Singh P. Eragamreddy S. Nguyen T. L. (2018). Twist1 activation in muscle progenitor cells causes muscle loss akin to cancer cachexia. *Dev. Cell.* 45 712.e6–725.e6. 29920276 10.1016/j.devcel.2018.05.026 PMC6054474

[B43] Pomies P. Blaquiere M. Maury J. Mercier J. Gouzi F. Hayot M. (2016). Involvement of the FoxO1/MuRF1/Atrogin-1 signaling pathway in the oxidative stress-induced atrophy of cultured chronic obstructive pulmonary disease myotubes. *PLoS One* 11:e0160092. 10.1371/journal.pone.0160092 27526027 PMC4987766

[B44] Qin Q. Xu Y. He T. Qin C. Xu J. (2012). Normal and disease-related biological functions of Twist1 and underlying molecular mechanisms. *Cell Res.* 22 90–106. 10.1038/cr.2011.144 21876555 PMC3351934

[B45] Reed S. A. Sandesara P. B. Senf S. M. Judge A. R. (2012). Inhibition of FoxO transcriptional activity prevents muscle fiber atrophy during cachexia and induces hypertrophy. *FASEB J.* 26 987–1000. 10.1096/fj.11-189977 22102632 PMC3289501

[B46] Reid J. Noble H. R. Porter S. Shields J. S. Maxwell A. P. (2013). A literature review of end-stage renal disease and cachexia: understanding experience to inform evidence-based healthcare. *J. Ren. Care* 39 47–51. 10.1111/j.1755-6686.2013.00341.x 23432742

[B47] Rigas J. R. Schuster M. Orlov S. V. Milovanovic B. Prabhash K. Smith J. T. (2010). Efect of ALD518, a humanized anti-IL-6 antibody, on lean body mass loss and symptoms in patients with advanced non-small cell lung cancer (NSCLC): results of a phase II randomized, double-blind safety and efficacy trial. *J. Clin. Oncol.* 28 (15_Suppl.):7622 10.1200/jco.2010.28.15_suppl.7622

[B48] Rohwedel J. Horak V. Hebrok M. Fuchtbauer E. M. Wobus A. M. (1995). M-twist expression inhibits mouse embryonic stem cell-derived myogenic differentiation in vitro. *Exp. Cell Res.* 220 92–100. 10.1006/excr.1995.1295 7664846

[B49] Rolfe D. F. Brown G. C. (1997). Cellular energy utilization and molecular origin of standard metabolic rate in mammals. *Physiol. Rev.* 77 731–758. 10.1152/physrev.1997.77.3.731 9234964

[B50] Scherbakov N. Von Haehling S. Anker S. D. Dirnagl U. Doehner W. (2013). Stroke induced Sarcopenia: muscle wasting and disability after stroke. *Int. J. Cardiol.* 170 89–94. 10.1016/j.ijcard.2013.10.031 24231058

[B51] Scicchitano B. M. Dobrowolny G. Sica G. Musaro A. (2018). Molecular insights into muscle homeostasis, atrophy and wasting. *Curr. Genomics* 19 356–369. 10.2174/1389202919666180101153911 30065611 PMC6030854

[B52] Sengupta A. Molkentin J. D. Yutzey K. E. (2009). FoxO transcription factors promote autophagy in cardiomyocytes. *J. Biol. Chem.* 284 28319–28331. 10.1074/jbc.m109.024406 19696026 PMC2788882

[B53] Souza T. A. Chen X. Guo Y. Sava P. Zhang J. Hill J. J. (2008). Proteomic identification and functional validation of activins and bone morphogenetic protein 11 as candidate novel muscle mass regulators. *Mol. Endocrinol.* 22 2689–2702. 10.1210/me.2008-0290 18927237 PMC5419403

[B54] Stitt T. N. Drujan D. Clarke B. A. Panaro F. Timofeyva Y. Kline W. O. (2004). The IGF-1/PI3K/Akt pathway prevents expression of muscle atrophy-induced ubiquitin ligases by inhibiting FOXO transcription factors. *Mol. Cell.* 14 395–403. 10.1016/s1097-2765(04)00211-4 15125842

[B55] Strassmann G. Masui Y. Chizzonite R. Fong M. (1993). Mechanisms of experimental cancer cachexia. Local involvement of IL-1 in colon-26 tumor. *J. Immunol.* 150 2341–2345. 8450216

[B56] Tan B. H. Birdsell L. A. Martin L. Baracos V. E. Fearon K. C. (2009). Sarcopenia in an overweight or obese patient is an adverse prognostic factor in pancreatic cancer. *Clin. Cancer Res.* 15 6973–6979. 10.1158/1078-0432.ccr-09-1525 19887488

[B57] Thakur S. S. Swiderski K. Ryall J. G. Lynch G. S. (2018). Therapeutic potential of heat shock protein induction for muscular dystrophy and other muscle wasting conditions. *Philos. Trans. R. Soc. Lond. B Biol. Sci.* 373:20160528. 10.1098/rstb.2016.0528 29203713 PMC5717528

[B58] Thisse B. El Messal M. Perrin-Schmitt F. (1987). The twist gene: isolation of a *Drosophila* zygotic gene necessary for the establishment of dorsoventral pattern. *Nucleic Acids Res.* 15 3439–3453. 10.1093/nar/15.8.3439 3106932 PMC340740

[B59] Tisdale M. J. (2010). Reversing cachexia. *Cell* 142 511–512. 10.1016/j.cell.2010.08.004 20723750

[B60] Vernon A. E. LaBonne C. (2004). Tumor metastasis: a new twist on epithelial-mesenchymal transitions. *Curr. Biol.* 14 R719–R721. 15341765 10.1016/j.cub.2004.08.048

[B61] Villain C. Ecochard R. Genet L. Jean G. Kuentz F. Lataillade D. (2015). Impact of BMI variations on survival in elderly hemodialysis patients. *J. Ren. Nutr.* 25 488–493. 10.1053/j.jrn.2015.05.004 26139338

[B62] von Haehling S. Ebner N. Dos Santos M. R. Springer J. Anker S. D. (2017). Muscle wasting and cachexia in heart failure: mechanisms and therapies. *Nat. Rev. Cardiol.* 14 323–341. 10.1038/nrcardio.2017.51 28436486

[B63] Waddell D. S. Baehr L. M. Van Den Brandt J. Johnsen S. A. Reichardt H. M. Furlow J. D. (2008). The glucocorticoid receptor and FOXO1 synergistically activate the skeletal muscle atrophy-associated MuRF1 gene. *Am. J. Physiol. Endocrinol. Metab.* 295 E785–E797. 18612045 10.1152/ajpendo.00646.2007 PMC2652500

[B64] Wang S. M. Coljee V. W. Pignolo R. J. Rotenberg M. O. Cristofalo V. J. Sierra F. (1997). Cloning of the human twist gene: its expression is retained in adult mesodermally-derived tissues. *Gene* 187 83–92. 10.1016/s0378-1119(96)00727-5 9073070

[B65] White J. P. (2017). IL-6, cancer and cachexia: metabolic dysfunction creates the perfect storm. *Transl. Cancer Res.* 6 S280–S285. 30766805 10.21037/tcr.2017.03.52 PMC6372111

[B66] Wildi S. Kleeff J. Maruyama H. Maurer C. A. Buchler M. W. Korc M. (2001). Overexpression of activin A in stage IV colorectal cancer. *Gut* 49 409–417. 10.1136/gut.49.3.409 11511564 PMC1728425

[B67] Williams M. J. Sugatani T. Agapova O. A. Fang Y. Gaut J. P. Faugere M. C. (2018). The activin receptor is stimulated in the skeleton, vasculature, heart, and kidney during chronic kidney disease. *Kidney Int.* 93 147–158. 10.1016/j.kint.2017.06.016 28843411 PMC6628245

[B68] Wolf C. Thisse C. Stoetzel C. Thisse B. Gerlinger P. Perrin-Schmitt F. (1991). The M-twist gene of Mus is expressed in subsets of mesodermal cells and is closely related to the *Xenopus* X-twi and the *Drosophila* twist genes. *Dev. Biol.* 143 363–373. 10.1016/0012-1606(91)90086-i 1840517

[B69] Xu J. Li R. Workeneh B. Dong Y. Wang X. Hu Z. (2012). Transcription factor FoxO1, the dominant mediator of muscle wasting in chronic kidney disease, is inhibited by microRNA-486. *Kidney Int.* 82 401–411. 10.1038/ki.2012.84 22475820 PMC3393843

[B70] Xu Y. Lee D. K. Feng Z. Bu W. Li Y. Liao L. (2017). Breast tumor cell-specific knockout of Twist1 inhibits cancer cell plasticity, dissemination, and lung metastasis in mice. *Proc. Natl. Acad. Sci. U.S.A.* 114 11494–11499. 10.1073/pnas.1618091114 29073077 PMC5664488

[B71] Xu Y. Liao L. Zhou N. Theissen S. M. Liao X. H. Nguyen H. (2013). Inducible knockout of Twist1 in young and adult mice prolongs hair growth cycle and has mild effects on general health, supporting Twist1 as a preferential cancer target. *Am. J. Pathol.* 183 1281–1292. 10.1016/j.ajpath.2013.06.021 23906809 PMC3791869

[B72] Yang J. Mani S. A. Donaher J. L. Ramaswamy S. Itzykson R. A. Come C. (2004). Twist, a master regulator of morphogenesis, plays an essential role in tumor metastasis. *Cell* 117 927–939. 10.1016/j.cell.2004.06.006 15210113

[B73] Yuen H. F. Chan Y. P. Wong M. L. Kwok W. K. Chan K. K. Lee P. Y. (2007). Upregulation of Twist in oesophageal squamous cell carcinoma is associated with neoplastic transformation and distant metastasis. *J. Clin. Pathol.* 60 510–514. 10.1136/jcp.2006.039099 16822877 PMC1994533

[B74] Zhang A. Li M. Wang B. Klein J. D. Price S. R. Wang X. H. (2018). miRNA-23a/27a attenuates muscle atrophy and renal fibrosis through muscle-kidney crosstalk. *J. Cachexia Sarcopenia Muscle* 9 755–770. 10.1002/jcsm.12296 29582582 PMC6104113

[B75] Zhang L. Rajan V. Lin E. Hu Z. Han H. Q. Zhou X. (2011). Pharmacological inhibition of myostatin suppresses systemic inflammation and muscle atrophy in mice with chronic kidney disease. *FASEB J.* 25 1653–1663. 10.1096/fj.10-176917 21282204 PMC3079306

[B76] Zhong X. Pons M. Poirier C. Jiang Y. Liu J. Sandusky G. E. (2019). The systemic activin response to pancreatic cancer: implications for effective cancer cachexia therapy. *J. Cachexia Sarcopenia Muscle* 10 1083–1101. 10.1002/jcsm.12461 31286691 PMC6818463

[B77] Zhou X. Wang J. L. Lu J. Song Y. Kwak K. S. Jiao Q. (2010). Reversal of cancer cachexia and muscle wasting by ActRIIB antagonism leads to prolonged survival. *Cell* 142 531–543. 10.1016/j.cell.2010.07.011 20723755

